# Paroxetine ameliorates lipopolysaccharide-induced microglia activation via differential regulation of MAPK signaling

**DOI:** 10.1186/1742-2094-11-47

**Published:** 2014-03-12

**Authors:** Rong-Pei Liu, Ming Zou, Jian-Yong Wang, Juan-Juan Zhu, Jun-Mei Lai, Li-Li Zhou, Song-Fang Chen, Xiong Zhang, Jian-Hong Zhu

**Affiliations:** 1 Department of Neurology & Geriatrics, the Second Affiliated Hospital, Wenzhou Medical University, Wenzhou, Zhejiang 325000, China; 2 Department of Preventive Medicine, Wenzhou Medical University, Wenzhou, Zhejiang 325035, China

**Keywords:** Paroxetine, Microglia, Lipopolysaccharide, Neuroinflammation, MAPK

## Abstract

**Background:**

Paroxetine, a selective serotonin reuptake inhibitor for counteracting depression, has been recently suggested as having a role in prevention of dopaminergic neuronal degeneration in substantia nigra, a hallmark of Parkinson’s disease (PD). The pathogenesis of this type of neurological disorders often involves the activation of microglia and associated inflammatory processes. Thus in this study we aimed to understand the role of paroxetine in microglia activation and to elucidate the underlying mechanism(s).

**Methods:**

BV2 and primary microglial cells were pretreated with paroxetine and stimulated with lipopolysaccharide (LPS). Cells were assessed for the responses of pro-inflammatory mediator and cytokines, and the related signaling pathways were evaluated and analyzed in BV2 cells.

**Results:**

Paroxetine significantly inhibited LPS-induced production of nitric oxide (NO) and pro-inflammatory cytokines such as TNF-α and IL-1β. Further analysis showed inducible nitric oxide synthase (iNOS) and mRNA expression of TNF-α and IL-1β were attenuated by paroxetine pretreatment. Analyses in signaling pathways demonstrated that paroxetine led to suppression of LPS-induced JNK1/2 activation and baseline ERK1/2 activity, but had little effect on the activation of p38 and p65/NF-κB. Interference with specific inhibitors revealed that paroxetine-mediated suppression of NO production was via JNK1/2 pathway while the cytokine suppression was via both JNK1/2 and ERK1/2 pathways. Furthermore, conditioned media culture showed that paroxetine suppressed the microglia-mediated neurotoxicity.

**Conclusions:**

Paroxetine inhibits LPS-stimulated microglia activation through collective regulation of JNK1/2 and ERK1/2 signaling. Our results indicate a potential role of paroxetine in neuroprotection via its anti-neuroinflammatory effect besides targeting for depression.

## Introduction

Parkinson’s disease (PD) is the second most common neurodegenerative disease characterized by a dramatic loss of dopaminergic neurons in substantia nigra. Although the etiology of PD and the underlying mechanisms for disease development remain incompletely understood, increasing evidence has suggested that inflammatory processes play a key role in the pathogenesis of PD [1-3]. Microglia are the resident macrophages of the central nervous system and act as the prime effector cells in mediating neuroinflammation [4,5]. It has been suggested that inflammatory mediators such as nitric oxide (NO), TNF-α, and IL-1β derived from microglia are involving in the progression of neuronal cell death in PD [6,7]. Indeed, lipopolysaccharide (LPS) as an inflammation elicitor has often been used to generate phenotypes of PD in animals [8,9]. Therefore, modulation of microglial activation and its production of pro-inflammatory mediators and cytokines would be a promising strategy to alleviate the progression of PD.

Paroxetine, a selective serotonin reuptake inhibitor, is often used as a first-line treatment in the treatment of depression because of its fewer side effects and lower toxicity compared with other antidepressants [10]. Considering depression is one of the most common non-motor symptoms of PD, occurring in approximately 35% of these patients [11], paroxetine has been clinically tested as a safe and effective drug to treat PD-associated depression [12,13]. Interestingly, a recent study disclosed that paroxetine can prevent the degeneration of nigrostriatal dopaminergic neurons by inhibiting glial activation and brain inflammation in an MPTP-induced animal model of PD [14], suggesting that paroxetine may also contribute to the alleviation of PD progression by inhibiting neuroinflammation, whereas the associating signaling mechanisms remain elusive. In the current study we devoted ourselves to further define the anti-inflammatory effect of paroxetine on microglia activation and, in particular, to dissect the underlying molecular mechanism(s).

## Materials and methods

### Reagents and cell culture

The BV2 microglial cells (gift of Dr. Zhu CQ, Fudan University) and SH-SY5Y cells (Cell Bank of Chinese Academy of Sciences, Shanghai, China) were grown in DMEM (Invitrogen, Grand Island, NY, USA) supplemented with 10% FBS (Invitrogen, Grand Island, NY, USA), penicillin (100 U/ml)/streptomycin (100 μg/ml) (Solarbio Science and Technology, Beijing, China). Cells were maintained in a humidified incubator at 37°C with 5% CO_2_. LPS and paroxetine were purchased from Sigma (St. Louis, MO, USA). BV2 cells were seeded at a density of 1 × 10^5^ cells/well in a 12-well plate, and allowed to settle at 37°C for 24 hours followed by serum starvation overnight. Cells were pretreated with paroxetine, SP600125 (Beyotime, Shanghai, China) or U0126 (Cell Signaling, Boston, MA, USA) for 30 minutes before LPS (100 ng/ml) stimulation.

Primary microglial cells were prepared as previously described with slight modifications [15]. Briefly, cerebral cortices were isolated from Institute of Cancer Research (ICR) mice at postnatal day one to two. Meninges and blood vessels were removed completely in cold Hank’s buffered saline. Cortices were then minced with sterile scissors and digested with 0.25% Trypsin-EDTA solution (Invitrogen, Grand Island, NY, USA) for 20 minutes at 37°C. Trypsinization was stopped by adding an equal volume of culture medium, that is, DMEM-F-12 nutrient mixture (Invitrogen, Grand Island, NY, USA) supplemented with 10% FBS and penicillin (100 U/ml)/streptomycin (100 μg/ml), followed by an addition of deoxyribonuclease I (65 unit/ml of final concentration; Solarbio Science and Technology, Beijing, China). The dissociated cells were pelleted at 200 g for five minutes, resuspended in culture medium, repeatedly pipetted and then passed through a 100 μm pore mesh. Cells were seeded on poly-L-lysine (1 mg/mL)-coated flasks and cultured at 37°C with 5% CO_2_. The medium was replaced every four to five days after seeding. After 12 to 14 days, microglial cells were isolated from mixed glial cultures by vigorous shaking for four hours at 200 rpm at 37°C. Cells were then pelleted, resuspended in mixed glial-conditioned medium and seeded into 24-well plates at a density of 5 × 10^5^ cells/well. Cells were washed with PBS and replaced with fresh culture medium after one hour to remove non-adherent cells. After 24 hours of culture, the cells were starved overnight and proceeded to treatments. The purity of primary microglial cells in the culture was assessed with staining of Iba-1 antibody (Wako, Osaka, Japan) and Hoechst 33258 (Beyotime, Shanghai, China).

### Cell viability

Cell viability was determined by the tetrazolium salt 3-[4,5-dimethylthiazol-2-yl]-2,5-diphenyltetrazolium bromide (MTT; Sigma, St. Louis, MO, USA) assay [16]. BV2 and primary microglial cells were initially seeded into 96-well plates at a density of 1 × 10^4^ cells/well and 5 × 10^4^ cells/well, respectively. Following treatment, MTT (5 mg/ml in PBS) was added to each well and incubated at 37°C for four hours. The resulting formazan crystals were dissolved in dimethylsulfoxide (DMSO). The optical density was measured at 570 nm, and results are expressed as a percentage of surviving cells compared with the control.

### Determination of cytokine production

Medium TNF-α and IL-1β were measured using ELISA kits purchased from R&D Systems (Minneapolis, MN, USA) following the manufacturer’s instruction. Briefly, standards and samples were added to a 96-well ELISA plate precoated with biotinylated anti-TNF-α or anti-IL-1β antibody. After washing away unbound substances, an enzyme-linked polyclonal antibody specific for TNF-α or IL-1β was added to the wells and incubated for two hours. The wells were then washed four times and filled with the substrate solution for an incubation of 30 minutes. The reaction was terminated by the stop solution. Absorbance was read at 450 nm in a microplate reader. The concentration of each sample was calculated from the standard curve prepared using the cytokine standards.

### NO release assay

Medium nitrite was measured as an indicator of NO production [17]. In brief, 50 μl of supernatant was mixed with an equal volume of Griess reagent I, followed by an addition of another 50 μl of Griess reagent II (Beyotime, Shanghai, China) at room temperature. Absorbance was immediately measured at 540 nm. The samples were assayed in triplicate, and the concentration of each sample was calculated from a standard curve generated using sodium nitrite.

### RNA isolation and RT-PCR

Total RNA was extracted using TRIZOL reagent (Invitrogen, Grand Island, NY, USA), and reverse-transcribed to cDNA using a kit from Tiangen (Tianjin, China). TNF-α and IL-1β genes were amplified using the following primer pairs: TNF-α, 5′-CGTCAGCCGATTTGCTATCT-3′ and 5′-CGGACTCCGCAAAGTCTAAG-3′; IL-1β, 5′-GCTGCTTCCAAACCTT-3′ and 5′-AGGCCACAGGTATTTT-3′; β-actin, 5′-GTGGGGCGCCCCAGGCACCA-3′ and 5′-CTTCCTTAATGTCACGCACGATTTC-3′. PCR reaction was conducted as follows: an initial denaturation at 94°C for three minutes, 32 cycles of 94°C for 30 seconds, 48°C (IL-1β) or 60°C (TNF-α and β-actin) for 45 seconds, 72°C for 30 seconds, then a final extension at 72°C for five minutes. The products were separated on a 1.2% agarose gel containing ethidium bromide, and were visualized under a gel imaging system.

### Western blotting analysis

Cells were lysed in sample buffer containing 60 mM Tris-HCl, pH 6.8, 5% glycerol and 2% SDS. Cell lysates were then boiled for five minutes and protein concentration was measured using a BCA kit purchased from Beyotime (Shanghai, China). Samples were subject to Western blot analysis as previously described [18]. In brief, equal amount of proteins was loaded and separated on a 7 or 10% SDS-PAGE gel and transferred to a PVDF membrane, which was then blocked with 5% milk for one hour at room temperature. The membrane was incubated overnight at 4°C with primary antibody followed by a secondary horseradish peroxidase-conjugated antibody for one hour at room temperature. Blots were developed using enhanced chemiluminescence (LumiGLO® Reagent and Peroxide, Cell Signaling, Boston, MA, USA) according to the manufacturer’s protocol. Primary antibodies against iNOS, p-JNK1/2, p-p38, p-ERK1/2, p-p65, JNK1/2, p38, ERK1/2, p65, and β-actin, and secondary anti-rabbit or anti-mouse antibody were all purchased from Cell Signaling (Boston, MA, USA).

### Microglia conditioned media

Human SH-SY5Y cells were plated in 96-well plates at a density of 1 × 10^4^ cells per well and allowed to settle for 24 hours at 37°C before replacement with conditioned media. Culture media of BV2 cells with different treatments were collected as conditioned media and clarified by centrifugation at 20,000 × g for five minutes to remove cellular debris. The media were then transferred onto SH-SY5Y cells. The viability of SH-SY5Y cells was measured using the MTT assay as described above after 24 hours incubation.

### Statistical analysis

Data were performed by a one-way analysis of variance (ANOVA) with Dunnett’s test using the statistical package of Predictive Analytics Software 18.0 (PASW, version 18.0) for windows. Difference was considered significant when *P* < 0.05.

## Results

### Paroxetine reduces pro-inflammatory cytokines in LPS-stimulated BV2 cells

**Figure 1:**
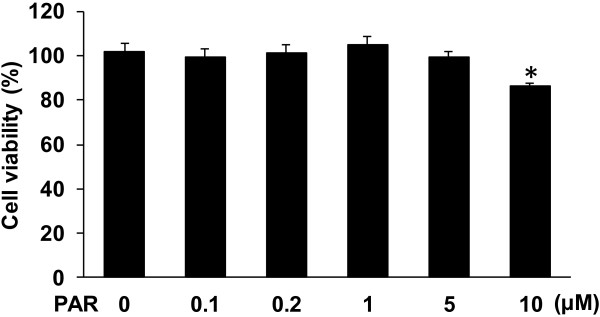
**Cell viability of BV2 cells treated with paroxetine.** Cells were treated with 0, 0.1, 0.2, 1, 5 or 10 μM of paroxetine for 24 hours. Cell viability was expressed as percentage of the control (0 μM), which was set as 100%. Values are means ± SE of three independent experiments. *P < 0.05 versus the control; PAR, paroxetine.

Prior to study the impact of paroxetine on LPS-induced microglial activation, we examined potential toxic effect of paroxetine on BV2 microglial cells. The results showed that cell viability was not different from the control (0 μM) following the treatment of paroxetine at 0.1, 0.2, 1 or 5 μM. The dose of 10 μM led to a 15.2% (*P* < 0.05) drop in cell viability compared with the control (Figure 1), which was then excluded in our following experiments.

**Figure 2:**
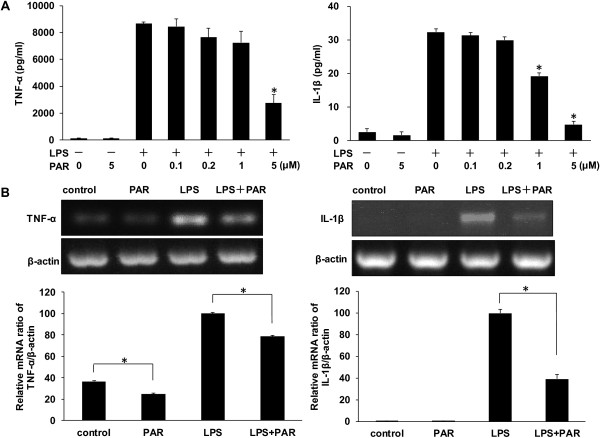
**Paroxetine attenuates lipopolysaccharide (LPS)-induced TNF-α and IL-1β in BV2 cells. (A)** Concentrations of TNF-α and IL-1β in culture media. BV2 cells were pretreated with paroxetine at 0, 0.1, 0.2, 1 or 5 μM for 30 minutes and then stimulated with LPS at 100 ng/ml for 24 hours. *P < 0.05 versus treated with LPS alone. **(B)** The mRNA expression of TNF-α and IL-1β. BV2 cells were pretreated with 5 μM paroxetine for 30 minutes followed by LPS treatment at 100 ng/mL for six hours. The mRNA levels of each cytokine were quantified and normalized with their respective β-actin. Each value was then expressed relative to the one treated with LPS alone, which was set as 100. *P < 0.05; values are means ± SE of three independent experiments. PAR, paroxetine; LPS, lipopolysaccharide.

To evaluate the impact of paroxetine on cytokine production following LPS stimulation in BV2 cells, we analyzed the release of two pro-inflammatory cytokines, TNF-α and IL-1β, in the media. BV2 cells were treated with LPS for 24 hours in the presence or absence of paroxetine. Paroxetine alone did not elicit marked alteration in the release of TNF-α or IL-1β, whereas LPS stimulation significantly elevated the levels of these two cytokines (Figure 2A). Pretreatment with paroxetine led to a dose-dependent inhibition on LPS-induced production of TNF-α and IL-1β. In particular, paroxetine at 5 μM led to a significant (*P* < 0.05) reduction by 68.3% and 85.3%, respectively, in TNF-α and IL-1β generation at 24 hours post LPS stimulation (Figure 2A). In order to understand the mechanism underlying the inhibitory effect of paroxetine on LPS-induced cytokine production, we analyzed the mRNA expression of TNF-α and IL-1β following LPS stimulation. Consistent with the cytokine release, LPS significantly up-regulated mRNA expression of TNF-α and IL-1β at 24 hours, which was in turn suppressed by 21.4% and 60.7%, respectively, with 5 μM of paroxetine pretreatment (Figure 2B). Paroxetine alone also slightly decreased the basal mRNA level of TNF-α, whereas the basal IL-1β level seems undetectable using our current PCR program (Figure 2B).

### Paroxetine suppresses LPS-induced NO production in BV2 cells

**Figure 3:**
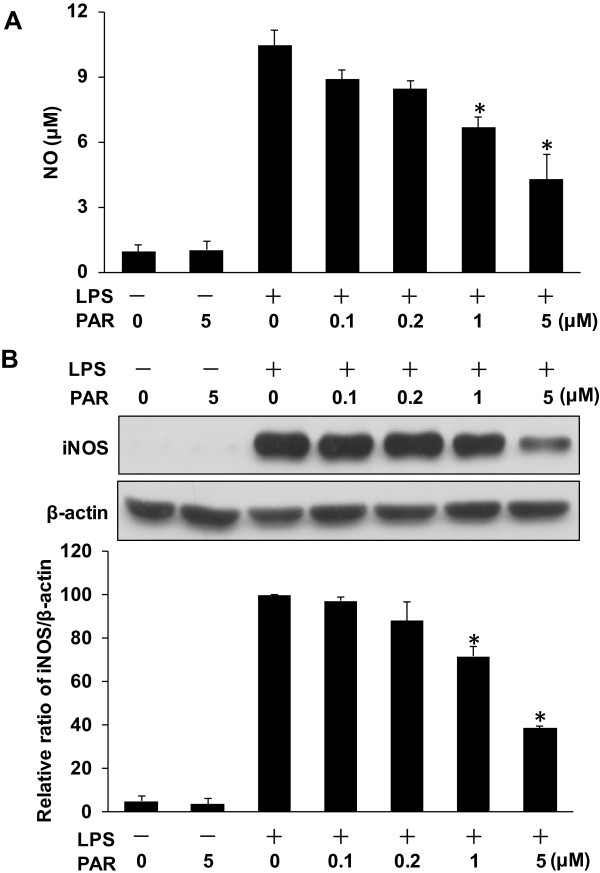
**Paroxetine inhibits lipopolysaccharide (LPS)-induced nitric oxide (NO) production and inducible nitric oxide synthase (iNOS) expression in BV2 cells.** Cells were pretreated with paroxetine at 0, 0.1, 0.2, 1 or 5 μM for 30 min and then stimulated with LPS at 100 ng/ml for 24 hours. **(A)** Measurement of nitrite in culture media as an indicator of NO production. **(B)** Western blot analysis of iNOS expression. The protein levels were quantified and normalized with their respective β-actin levels. Each value was then expressed relative to the one treated with LPS alone, which was set as 100. *P < 0.05 versus treated with LPS alone. Values are means ± SE of three independent experiments. PAR, paroxetine; LPS, lipopolysaccharide; NO, nitric oxide; iNOS, inducible nitric oxide synthase.

To assess whether paroxetine has an impact on NO release in microglial cells, we analyzed NO production following LPS stimulation. BV2 cells were treated with LPS for 24 hours in the presence or absence of paroxetine. As shown in Figure 3A, paroxetine alone did not lead to any change in NO production, whereas LPS significantly induced the generation of NO in BV2 cells. Pretreatment with paroxetine led to a dose-dependent inhibition on LPS-induced NO production by 15.1% at 0.1 μM, 19.1% at 0.2 μM, 36.2% (*P* < 0.05) at 1 μM, and 59.1% (*P* < 0.05) at 5 μM (Figure 3A). To understand the mechanism responsible for the paroxetine-mediated inhibition on LPS-induced NO production, we analyzed the expression of inducible nitric oxide synthase (iNOS) following LPS stimulation. Paroxetine alone did not change iNOS level, while LPS treatment significantly up-regulated iNOS expression. In line with the changes in NO production, pretreatment with paroxetine led to a dose-dependent suppression on LPS-induced iNOS expression by 2.9% at 0.1 μM, 12.0% at 0.2 μM, 28.4% (*P* < 0.05) at 1 μM, and 61.4% (*P* < 0.05) at 5 μM (Figure 3B).

### Paroxetine blocks LPS-induced JNK activation and attenuates baseline ERK1/2 activity in BV2 cells

**Figure 4:**
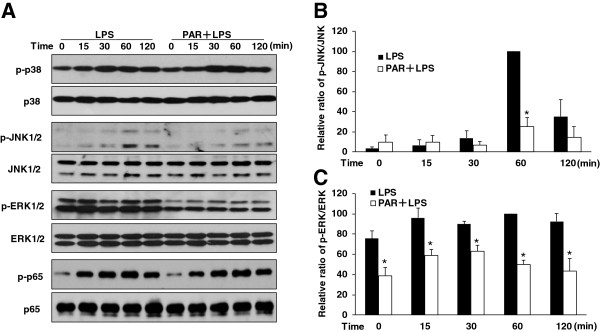
**Effect of paroxetine on lipopolysaccharide (LPS)-stimulated activation of MAPK and NF-κB in BV2 cells.** Cells were pretreated with 5 μM paroxetine for 30 minutes followed by the treatment of LPS at 100 ng/mL for 0, 15, 30, 60 or 120 minutes. **(A)** Representative images of Western blot for the activation of p38, JNK1/2, ERK1/2 and p65/NF-κB. The levels of p-JNK1/2 **(B)** and p-ERK1/2 **(C)** were quantified and normalized with their respective total JNK1/2 or Erk1/2 levels. Each value was then expressed relative to the one treated with LPS alone for 60 minutes, which was set as 100. *P < 0.05 versus treated with LPS alone within the same time point. Values are means ± SE of three independent experiments. PAR, paroxetine; LPS, lipopolysaccharide.

A number of studies have demonstrated that NF-κB and MAPKs have important roles in modulating the expression of pro-inflammatory cytokines and iNOS in LPS-stimulated microglia [19,20]. Therefore, we investigated the effect of paroxetine on the activity of p38, JNK, ERK1/2, and p65/NF-κB in BV2 cells following LPS stimulation. Paroxetine alone did not have any effect on the activation of these kinases except ERK1/2 which displayed a drastic drop (approximately 45%) in baseline phosphorylation upon 5 μM of paroxetine treatment (Figure 4A and C). Interestingly, LPS stimulation did not elicit activation of ERK1/2 but indeed induced marked activation of JNK1/2, p38, and p65/NF-κB in a time-dependent manner (Figure 4A). The peak of activation for each kinase varied, such as p38 peaked at 30 minutes post LPS stimulation, JNK1/2 and p65 peaked at one hour. Pretreatment with paroxetine in BV2 cells markedly blocked LPS-induced JNK1/2 activation, but showed little influence on the activation of p38 and p65 kinases (Figure 4A and B).

### Paroxetine inhibits LPS-induced microglial activation through JNK and ERK pathways

**Figure 5:**
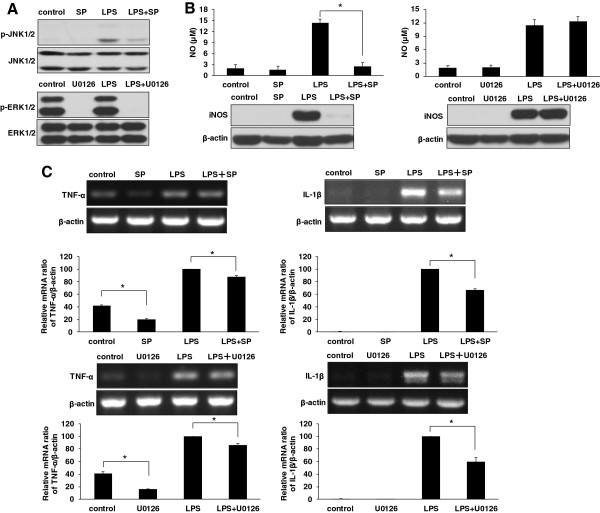
**Inhibition of JNK or ERK signaling on lipopolysaccharide (LPS)-mediated microglia activation. (A)** Inhibitory effect of SP600125 and U0126 on JNK1/2 and ERK1/2 activation. BV2 cells were treated with SP600125 (20 μM) or U0126 (10 μM) for 30 minutes prior to LPS treatment (100 ng/mL) for one hour. **(B)** Measurement of NO production in culture media (upper panel) and Western blot analysis of inducible nitric oxide synthase (iNOS) expression (lower panel). Cells were pretreated with SP600125 (20 μM) or U0126 (10 μM) for 30 minutes followed by stimulation of LPS (100 ng/mL) for 24 hours. **(C)** The mRNA expression of TNF-α and IL-1β. Cells were pretreated with SP600125 (20 μM) or U0126 (10 μM) for 30 minutes followed by stimulation of LPS (100 ng/mL) for six hours. The mRNA levels of each cytokine were quantified and normalized with their respective β-actin. Each value was then expressed relative to the one treated with LPS alone, which was set as 100. *P < 0.05. Values are means ± SE of three independent experiments. SP, SP600125. LPS, lipopolysaccharide.

Since paroxetine inhibited LPS-induced JNK activation as well as baseline ERK1/2 activity, we then asked whether the inhibitory effect of paroxetine on microglial activation is via JNK and (or) ERK pathways. We investigated the effect of specific JNK inhibitor SP600125 and specific ERK1/2 inhibitor U0126 on LPS-induced NO production and pro-inflammatory cytokines in BV2 cells. SP600125 and U0126 were firstly verified for their abilities to block JNK1/2 and ERK1/2 activation, respectively, in BV2 cells (Figure 5A). Pretreatment with SP600125 significantly suppressed LPS-induced NO production by 82.3%. In contrast, U0126 showed no effect on the NO production. In line with the regulation on NO production, LPS-induced iNOS expression was blocked by SP600125, but not by U0126 (Figure 5B). On the other hand, both SP600125 and U0126 blunted LPS-induced cytokine up-regulation. SP600125 pretreatment resulted in a significant reduction by 12.1% and 33.5% (*P* < 0.05), respectively, on LPS-induced TNF-α and IL-1β mRNA expression, while U0126 reduced the elevation of these two cytokines by 13.6% and 40.6% (*P* < 0.05), respectively (Figure 5C). Similar to paroxetine, SP600125 and U0126 also reduced the basal mRNA expression of TNF-α in BV2 cells (Figure 5C).

### Paroxetine relieves microglia-mediated neurotoxicity

**Figure 6:**
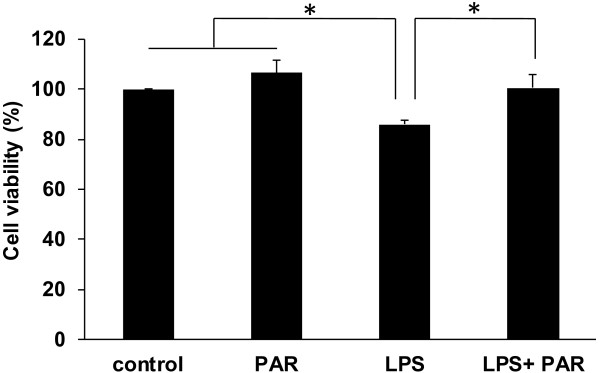
**Paroxetine relieves microglia-mediated neurotoxicity.** BV2 cells were first treated with lipopolysaccharide (LPS) (100 ng/mL) for 24 hours with or without 30 minutes of paroxetine pretreatment at 5 μM. The media were then collected as condition media and added to SH-SY5Y cells. After 24 hours incubation, cell viability of SH-SY5Y was assessed and expressed as percentage of the control, which was set as 100%. *P < 0.05. Values are means ± SE of three independent experiments. PAR, paroxetine; LPS, lipopolysaccharide.

Microglia upon activation could induce neuronal cell degeneration by releasing inflammatory mediators and cytokines [6,21,22]. We therefore investigated whether paroxetine contributes to the relief of activated microglia-induced neurotoxicity. The neuroblastoma cell line SH-SY5Y is often used in the cellular model of PD due to its dopaminergic ability [23,24]. As shown in Figure 6, conditioned media from LPS-stimulated, but not from paroxetine alone-treated, BV2 cells significantly (*P* < 0.05) increased cell death of SH-SY5Y cells. In contrast, the conditioned media from BV2 cells pretreated with paroxetine prior to LPS stimulation showed little neurotoxicity on SH-SY5Y cells (Figure 6), suggesting that paroxetine suppresses microglia-mediated neurotoxicity via reducing the expression of inflammatory mediators.

### Paroxetine suppresses LPS-stimulated pro-inflammatory cytokines and NO in primary microglial cells

**Figure 7:**
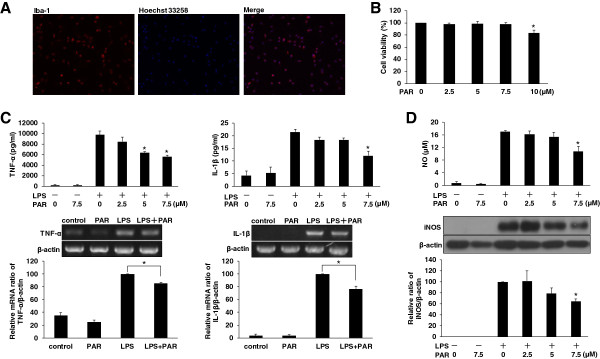
**Paroxetine suppresses the lipopolysaccharide (LPS)-stimulated pro-inflammatory cytokines and nitric oxide (NO) in primary microglial cells. (A)** Purity assessment of isolated primary microglial cells. Cells were immunostained with ani-Iba-1 antibody (red) and Hoechst 33258 for nuclei (blue). **(B)** Cell viability analysis. Cells were treated with 0, 2.5, 5, 7.5 or 10 μM of paroxetine for 24 hours. Cell viability was expressed relative to the control (0 μM), which was set as 100%. Values are means ± SE of three independent experiments. *P < 0.05 versus the control. **(C)** Effect of paroxetine on TNF-α and IL-1β productions. For cytokine release in media (the upper panel), cells were pretreated with paroxetine for 30 minutes and then stimulated with LPS at 100 ng/ml for 24 hours. *P < 0.05 versus treated with LPS alone. For mRNA expression (the lower panel), cells were pretreated with 7.5 μM paroxetine for 30 minutes followed by LPS treatment at 100 ng/mL for six hours. The mRNA levels of each cytokine were quantified and normalized with their respective β-actin. Each value was expressed relative to the one treated with LPS alone, which was set as 100. *P < 0.05; values are means ± SE of four independent experiments. **(D)** Effect of paroxetine on NO production (the upper panel) and inducible nitric oxide synthase (iNOS) expression (the lower panel). Cells were pretreated with paroxetine for 30 minutes and then stimulated with LPS at 100 ng/ml for 24 hours. The iNOS protein levels were quantified and normalized with their respective β-actin. Each value was expressed relative to the one treated with LPS alone, which was set as 100. *P < 0.05 versus treated with LPS alone. Values are means ± SE of four independent experiments. PAR, paroxetine; LPS, lipopolysaccharide; NO, nitric oxide; iNOS, inducible nitric oxide synthase.

Primary microglial cells were isolated to repeat the inhibitory effect of paroxetine on the cytokine and NO production as observed in BV2 cells. Purity assessment of the isolation displayed more than 98% of the cells with positive staining (Figure 7A). We then evaluated the effect of paroxetine on the survival of primary microglial cells. Cell viability was not different from the control (0 μM) following the treatment of paroxetine at 2.5, 5 or 7.5 μM, while the dose of 10 μM led to a 16.1% (*P* < 0.05) decrease in cell viability (Figure 7B) and then was excluded for the following experiments.

As expected LPS stimulation of primary microglial cells led to a significant increase in cytokine release and NO production after 24 hours. Pretreatment of primary cells with paroxetine significantly inhibited the LPS-induced TNF-α, IL-1β and NO productions in a dose-dependent manner, while paroxetine alone did not apparently alter the level of these mediators (Figure 7C and D). In particular, paroxetine at 7.5 μM led to a significant (*P* < 0.05) reduction by 45.7, 43.9 and 36.7%, respectively, in TNF-α, IL-1β and NO productions at 24 hours post LPS stimulation. Further analysis showed that the LPS-induced mRNA expression of TNF-α and IL-1β at six hours was reduced by 14.4% and 23.3%, respectively, with 7.5 μM of paroxetine pretreatment (Figure 7C). Similar to BV2 cells, paroxetine alone also slightly decreased the basal mRNA level of TNF-α, whereas the basal IL-1β level appeared under our detection limit. LPS-stimulated iNOS expression was dose-dependently attenuated by paroxetine with an inhibition of 36% at the dose of 7.5 μM (Figure 7D).

## Discussion

Microglia, an immune-like cell of the brain, plays an important role in inflammatory responses in the central nervous system. Activated microglia secrete large amounts of neurotoxic factors, such as NO, TNF-α and IL-1β. Recent studies have shown that these cytotoxic factors play a critical role in the pathogenesis of brain injury and neurodegenerative disorders such as PD and Alzheimer’s disease [25], and also affect complex central nervous system functions such as cognition, sleep and depression [26-29]. Thus, inhibition of microglia activation serves as a key mechanism in the treatment of inflammation-associated neurological disorders. The current study demonstrated an inhibitory role of paroxetine in microglia activation stimulated by LPS and elucidated the underlying molecular mechanism, that is, paroxetine suppresses LPS-induced NO production via mediation of JNK1/2 activation, and inhibits pro-inflammatory cytokines such as TNF-α and IL-1β via collective regulation of JNK1/2 activation and baseline ERK1/2 activity. Meanwhile, we observed that paroxetine reduced BV2 microglia-mediated neurotoxicity in line with the view that reduction of microglia releasing excessive amount of neurotoxic mediators is neuroprotective [30,31].

Paroxetine exhibited comparable inhibitory effects on NO and cytokine productions in BV2 cell lines and primary microglial cells. NO is generated from L-arginine by three different isoforms of NOS, including endothelial NOS, neuronal NOS and iNOS [32]. Expression of iNOS occurs primarily in astrocytes and microglia in response to extracellular stimuli including LPS, IL-1β, IFN-γ, and TNF-α [33,34]. Excessive release of NO by activated microglia leads to formation of peroxynitrite by reacting with superoxide, which intoxicates cells by disturbing mitochondrial respiration, reacting with cellular molecules [35]. Our results showed that paroxetine suppressed the LPS-elicited iNOS up-regulation in both types of cells and thereby prevented the increase of NO production. The basal NO level was not reduced by paroxetine treatment, most likely due to the minimum baseline iNOS expression. For cytokines, paroxetine markedly inhibited LPS-induced elevation in both mRNA expression and peptide release of TNF-α and IL-1β in BV2 and primary microglial cells. Interestingly the paroxetine-induced baseline change of TNF-α in peptide release and mRNA expression appeared in a discrepancy as the basal release of TNF-α in media did not differ but its basal mRNA expression was to some extent reduced by paroxetine, suggesting a differential response of microglial TNF-α mRNA translating to the release of peptide under normal and stressed (that is with LPS stimulation) conditions. The situation is unclear regarding IL-1β as its basal mRNA expression was undetectable under our PCR condition. Tynan *et al*. recently screened a set of antidepressants mainly focusing on the comparison of immunomodulatory effects between selective serotonin reuptake inhibitors and serotonin-norepinephrine reuptake inhibitors, where an inhibitory effect of paroxetine against LPS-stimulated production of NO and TNF-α was also mentioned; however, this was without further exploration on paroxetine and associated signal wirings [36]. As far as drug dosage is concerned, recommended therapeutic range of paroxetine reaches a level between 0.19 and 0.32 μM in serum, and the level of psychotropic drugs is usually detected 10 to 40 times higher in brain than in blood [37]. Therefore, the 0.1 to 7.5 μM paroxetine used in this study is comparable to the putative level of therapeutic doses in brain, and should be safe for other tissues when dosage is administered therapeutically.

NF-κB and MAPK family including JNK, p38 and ERK are key regulators involved in the production of cytokines and mediators associated with the pathogenesis of inflammatory processes [38-40]. Indeed, LPS induced NF-κB activation as manifested by the phosphorylation of p65 subunit, as well as p38 and JNK1/2 activation in BV2 cells. However, ERK1/2 activity was not elevated following LPS stimulation as documented in several other studies [41,42]. Pretreatment with paroxetine did not apparently change LPS-induced p65 and p38 activation, demonstrating that the anti-inflammatory property of paroxetine does not rely on NF-κB and p38 signaling. On the other hand, baseline ERK1/2 activity and LPS-induced JNK1/2 activation were blunted by paroxetine pre-administration, suggesting paroxetine-mediated anti-microglia activation is potentially via inhibition of JNK1/2 and (or) ERK1/2 activities. These differential regulations indicate that paroxetine preferentially targets the upstream of JNK and ERK signaling. Unfortunately we cannot provide further clues at this point due to the complexity and frequent crosstalk in the MAPK network. Instead, we analyzed how mediation of JNK and ERK signaling by paroxetine contributes to the inhibition of microglia activation.

First, with regard to NO production, inhibition of JNK1/2 signaling by a specific inhibitor SP600125 led to nearly complete abolishment of LPS-induced iNOS expression and NO production, whereas inhibition of ERK1/2 signaling by U0126 displayed no effect, suggesting iNOS expression is induced mainly through JNK1/2 signaling. Indeed, suppression of iNOS induction and NO production in reactive microglia by JNK1/2 inhibitors has been consistently reported [43,44], while the role of ERK seems a bit controversial as both inhibition and no impact by ERK1/2 inhibitors have been reported [43,45]. Importantly, the data above demonstrated that paroxetine-mediated suppression of NO production is via mediation of JNK1/2 activation, but not through ERK1/2 signaling. Compared with paroxetine, SP600125 displayed a stronger inhibitory effect to iNOS expression and NO production, which is apparently due to SP600125 being a more potent inhibitor for JNK1/2 activity.

As far as pro-inflammatory cytokines are concerned, both inhibition of JNK1/2 by SP600125 and inhibition of ERK1/2 by U0126 resulted in a reduction of LPS-stimulated TNF-α or IL-1β production. Data analysis showed that the reduction of LPS-elicited cytokine production by paroxetine (21.4% and 60.7%, respectively for TNF-α and IL-1β) was smaller than the sum (25.6% and 74.1%, respectively), but larger than the individual values of the inhibition rates by JNK1/2 inhibitor SP600125 (12.1% and 33.5%, respectively) and ERK1/2 inhibitor U0126 (13.6% and 40.6%, respectively), demonstrating that paroxetine suppresses LPS-induced cytokine production collectively via JNK1/2 and ERK1/2 signaling, but not likely through a single pathway. We also tried to simultaneously block JNK1/2 and ERK1/2 activities to further determine whether other pathways are involved in the action of paroxetine. However, this effort was prevented due to a sharp decrease in cell number following the addition of both SP600125 and U0126 (data not shown), indicating the presence of some activity from at least one of the pathways is required for the BV2 cell survival. On the other hand, paroxetine-mediated inhibition of baseline cytokine production seems solely via inhibition of ERK1/2 signaling since ERK1/2 but not JNK1/2 baseline activity was suppressed by paroxetine. Indeed, the inhibition rate of basal TNF-α production with paroxetine (11.9%) did not exceed that with U0126 (24.3%), a more potent ERK1/2 inhibitor. Interestingly, a fellow serotonin reuptake inhibitor, fluoxetine, was also reported to inhibit LPS-mediated microglia activation, but through regulation of NF-κB and p38 activation [39], suggesting different signaling mechanisms were involved in antidepressant mediated anti-neuroinflammation.

## Conclusions

**Figure 8:**
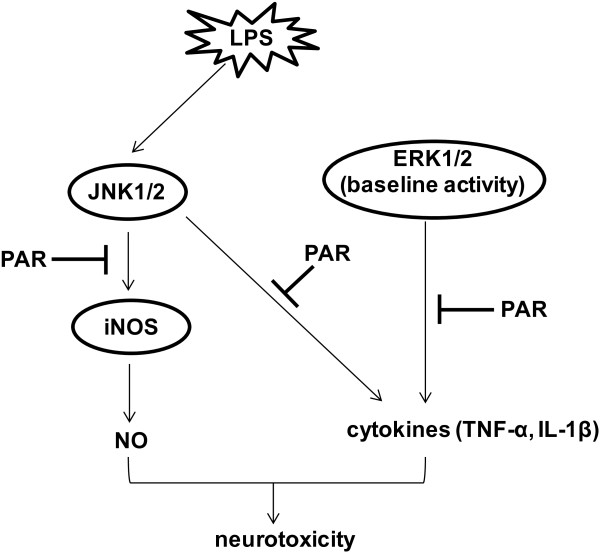
**Schematic illustration of paroxetine-mediated suppression of neuroinflammation.** PAR, paroxetine; LPS, lipopolysaccharide; NO, nitric oxide; iNOS, inducible nitric oxide synthase; ➔, lead to/activate; ▬▌, inhibit.

In summary, the present study demonstrated the inhibitory role of paroxetine in LPS-induced neuroinflammation and dissected the underlying molecular mechanisms, that is, paroxetine inhibits iNOS induction and NO generation by suppressing JNK1/2 activation, and attenuates cytokine production by collectively inhibiting JNK1/2 activation and baseline ERK1/2 activity (Figure 8). Since paroxetine is originally set as an antidepressant, our results provide further evidence to the point of view that depression involves neuroinflammatory processes [36,46]. Given the pathogenic role of inflammation in PD together with the previous report showing paroxetine-mediated prevention of neuronal degeneration in substantia nigra [14], we cautiously suggest that paroxetine may possibly be helpful in alleviating PD progression.

## Abbreviations

DMEM: Dulbecco’s modified Eagle medium; ERK: Extracellular signal-regulated kinase; FBS: Fetal bovine serum; ICR: Imprinting control region; IL-1β: Interleukin 1β; iNOS: Inducible nitric oxide synthase; JNK: c-jun N-terminal kinase; LPS: Lipopolysaccharide; MAPK: Mitogen-activated protein kinase; NF-κB: Nuclear factor κB; NO: nitric oxide; PBS: Phosphate buffered saline; PD: Parkinson’s disease; PCR: Polymerase chain reaction; TNF-α: Tumor necrosis factor alpha.

## Competing interests

The authors declare no competing interest.

## Authors’ contributions

RPL, MZ, JYW, JJZ, JML, LLZ and SFC performed the experiments; XZ and JHZ designed the study; RPL and JHZ wrote the manuscript. All authors read and approved the final manuscript.

## References

[B1] McGeer PL McGeer EG Inflammation and neurodegeneration in Parkinson’s disease Parkinsonism Relat Disord 2004 10 Suppl 1 S3 S7 15109580 10.1016/j.parkreldis.2004.01.005

[B2] Block ML Zecca L Hong JS Microglia-mediated neurotoxicity: uncovering the molecular mechanisms Nat Rev Neurosci 2007 8 57 69 10.1038/nrn2038 17180163

[B3] Appel SH Inflammation in Parkinson’s disease: cause or consequence? Mov Disord 2012 27 1075 1077 10.1002/mds.25111 22806694

[B4] Amor S Puentes F Baker D van der Valk P Inflammation in neurodegenerative diseases Immunology 2010 129 154 169 10.1111/j.1365-2567.2009.03225.x 20561356 PMC2814458

[B5] Griffiths MR Gasque P Neal JW The multiple roles of the innate immune system in the regulation of apoptosis and inflammation in the brain J Neuropathol Exp Neurol 2009 68 217 226 10.1097/NEN.0b013e3181996688 19225414

[B6] Saijo K Winner B Carson CT Collier JG Boyer L Rosenfeld MG Gage FH Glass CK A Nurr1/CoREST pathway in microglia and astrocytes protects dopaminergic neurons from inflammation-induced death Cell 2009 137 47 59 10.1016/j.cell.2009.01.038 19345186 PMC2754279

[B7] Glass CK Saijo K Winner B Marchetto MC Gage FH Mechanisms underlying inflammation in neurodegeneration Cell 2010 140 918 934 10.1016/j.cell.2010.02.016 20303880 PMC2873093

[B8] Hsieh PF Chia LG Ni DR Cheng LJ Ho YP Tzeng SF Chang MH Hong JS Behavior, neurochemistry and histology after intranigral lipopolysaccharide injection Neuroreport 2002 13 277 280 10.1097/00001756-200203040-00006 11930122

[B9] Hunter RL Cheng B Choi DY Liu M Liu S Cass WA Bing G Intrastriatal lipopolysaccharide injection induces parkinsonism in C57/B6 mice J Neurosci Res 2009 87 1913 1921 10.1002/jnr.22012 19224579 PMC2692550

[B10] Barbui C Hotopf M Freemantle N Boynton J Churchill R Eccles MP Geddes JR Hardy R Lewis G Mason JM Selective serotonin reuptake inhibitors versus tricyclic and heterocyclic antidepressants: comparison of drug adherence Cochrane Database Syst Rev 2000 4 CD002791 11034764 10.1002/14651858.CD002791

[B11] Aarsland D Pahlhagen S Ballard CG Ehrt U Svenningsson P Depression in Parkinson disease–epidemiology, mechanisms and management Nat Rev Neurol 2012 8 35 47 22198405 10.1038/nrneurol.2011.189

[B12] Ceravolo R Nuti A Piccinni A Dell’Agnello G Bellini G Gambaccini G Dell’Osso L Murri L Bonuccelli U Paroxetine in Parkinson’s disease: effects on motor and depressive symptoms Neurology 2000 55 1216 1218 10.1212/WNL.55.8.1216 11071504

[B13] Tesei S Antonini A Canesi M Zecchinelli A Mariani CB Pezzoli G Tolerability of paroxetine in Parkinson’s disease: a prospective study Mov Disord 2000 15 986 989 10.1002/1531-8257(200009)15:5<986::AID-MDS1034>3.0.CO;2-I 11009210

[B14] Chung YC Kim SR Jin BK Paroxetine prevents loss of nigrostriatal dopaminergic neurons by inhibiting brain inflammation and oxidative stress in an experimental model of Parkinson’s disease J Immunol 2010 185 1230 1237 10.4049/jimmunol.1000208 20566832

[B15] Song X Shapiro S Goldman DL Casadevall A Scharff M Lee SC Fcgamma receptor I- and III-mediated macrophage inflammatory protein 1alpha induction in primary human and murine microglia Infect Immun 2002 70 5177 5184 10.1128/IAI.70.9.5177-5184.2002 12183568 PMC128255

[B16] Zhu JH Lei XG Double null of selenium-glutathione peroxidase-1 and copper, zinc-superoxide dismutase enhances resistance of mouse primary hepatocytes to acetaminophen toxicity Exp Biol Med (Maywood) 2006 231 545 552 16636302 10.1177/153537020623100508

[B17] Wilms H Sievers J Rickert U Rostami-Yazdi M Mrowietz U Lucius R Dimethylfumarate inhibits microglial and astrocytic inflammation by suppressing the synthesis of nitric oxide, IL-1beta, TNF-alpha and IL-6 in an *in vitro* model of brain inflammation J Neuroinflammation 2010 7 30 10.1186/1742-2094-7-30 20482831 PMC2880998

[B18] Zhu JH Chen CL Flavahan S Harr J Su B Flavahan NA Cyclic stretch stimulates vascular smooth muscle cell alignment by redox-dependent activation of Notch3 Am J Physiol Heart Circ Physiol 2011 300 H1770 H1780 10.1152/ajpheart.00535.2010 21169401 PMC3094076

[B19] Kim SH Smith CJ Van Eldik LJ Importance of MAPK pathways for microglial pro-inflammatory cytokine IL-1 beta production Neurobiol Aging 2004 25 431 439 10.1016/S0197-4580(03)00126-X 15013563

[B20] Jack CS Arbour N Manusow J Montgrain V Blain M McCrea E Shapiro A Antel JP TLR signaling tailors innate immune responses in human microglia and astrocytes J Immunol 2005 175 4320 4330 16177072 10.4049/jimmunol.175.7.4320

[B21] Kaushal V Schlichter LC Mechanisms of microglia-mediated neurotoxicity in a new model of the stroke penumbra J Neurosci 2008 28 2221 2230 18305255 10.1523/JNEUROSCI.5643-07.2008 PMC6671848

[B22] Novarino G Fabrizi C Tonini R Denti MA Malchiodi-Albedi F Lauro GM Sacchetti B Paradisi S Ferroni A Curmi PM Breit SN Mazzanti M Involvement of the intracellular ion channel CLIC1 in microglia-mediated beta-amyloid-induced neurotoxicity J Neurosci 2004 24 5322 5330 10.1523/JNEUROSCI.1170-04.2004 15190104 PMC6729296

[B23] Biedler JL Roffler-Tarlov S Schachner M Freedman LS Multiple neurotransmitter synthesis by human neuroblastoma cell lines and clones Cancer Res 1978 38 3751 3757 29704

[B24] Farooqui SM Induction of adenylate cyclase sensitive dopamine D2-receptors in retinoic acid induced differentiated human neuroblastoma SHSY-5Y cells Life Sci 1994 55 1887 1893 10.1016/0024-3205(94)00520-6 7990648

[B25] Tambuyzer BR Ponsaerts P Nouwen EJ Microglia: gatekeepers of central nervous system immunology J Leukoc Biol 2009 85 352 370 19028958 10.1189/jlb.0608385

[B26] Dantzer R O’Connor JC Freund GG Johnson RW Kelley KW From inflammation to sickness and depression: when the immune system subjugates the brain Nat Rev Neurosci 2008 9 46 56 10.1038/nrn2297 18073775 PMC2919277

[B27] McAfoose J Baune BT Evidence for a cytokine model of cognitive function Neurosci Biobehav Rev 2009 33 355 366 10.1016/j.neubiorev.2008.10.005 18996146

[B28] Menza M Dobkin RD Marin H Mark MH Gara M Bienfait K Dicke A Kusnekov A The role of inflammatory cytokines in cognition and other non-motor symptoms of Parkinson’s disease Psychosomatics 2010 51 474 479 21051678 10.1176/appi.psy.51.6.474 PMC2987579

[B29] Suzuki E Yagi G Nakaki T Kanba S Asai M Elevated plasma nitrate levels in depressive states J Affect Disord 2001 63 221 224 10.1016/S0165-0327(00)00164-6 11246099

[B30] McGeer PL Itagaki S Boyes BE McGeer EG Reactive microglia are positive for HLA-DR in the substantia nigra of Parkinson’s and Alzheimer’s disease brains Neurology 1988 38 1285 1291 10.1212/WNL.38.8.1285 3399080

[B31] Gao HM Hong JS Why neurodegenerative diseases are progressive: uncontrolled inflammation drives disease progression Trends Immunol 2008 29 357 365 10.1016/j.it.2008.05.002 18599350 PMC4794280

[B32] Nathan C Xie QW Nitric oxide synthases: roles, tolls, and controls Cell 1994 78 915 918 10.1016/0092-8674(94)90266-6 7522969

[B33] Shen S Yu S Binek J Chalimoniuk M Zhang X Lo SC Hannink M Wu J Fritsche K Donato R Sun GY Distinct signaling pathways for induction of type II NOS by IFNgamma and LPS in BV-2 microglial cells Neurochem Int 2005 47 298 307 10.1016/j.neuint.2005.03.007 15955597

[B34] Moss DW Bates TE Activation of murine microglial cell lines by lipopolysaccharide and interferon-gamma causes NO-mediated decreases in mitochondrial and cellular function Eur J Neurosci 2001 13 529 538 10.1046/j.1460-9568.2001.01418.x 11168560

[B35] Pacher P Beckman JS Liaudet L Nitric oxide and peroxynitrite in health and disease Physiol Rev 2007 87 315 424 10.1152/physrev.00029.2006 17237348 PMC2248324

[B36] Tynan RJ Weidenhofer J Hinwood M Cairns MJ Day TA Walker FR A comparative examination of the anti-inflammatory effects of SSRI and SNRI antidepressants on LPS stimulated microglia Brain Behav Immun 2012 26 469 479 10.1016/j.bbi.2011.12.011 22251606

[B37] Baumann P Ulrich S Eckermann G Gerlach M Kuss HJ Laux G Muller-Oerlinghausen B Rao ML Riederer P Zernig G Hiemke C The AGNP-TDM Expert Group Consensus Guidelines: focus on therapeutic monitoring of antidepressants Dialogues Clin Neurosci 2005 7 231 247 16156382 10.31887/DCNS.2005.7.3/pbaumann PMC3181735

[B38] Baldwin AS Jr The NF-kappa B and I kappa B proteins: new discoveries and insights Annu Rev Immunol 1996 14 649 683 10.1146/annurev.immunol.14.1.649 8717528

[B39] Liu D Wang Z Liu S Wang F Zhao S Hao A Anti-inflammatory effects of fluoxetine in lipopolysaccharide(LPS)-stimulated microglial cells Neuropharmacology 2011 61 592 599 10.1016/j.neuropharm.2011.04.033 21575647

[B40] Jung HW Chung YS Kim YS Park YK Celastrol inhibits production of nitric oxide and proinflammatory cytokines through MAPK signal transduction and NF-kappaB in LPS-stimulated BV-2 microglial cells Exp Mol Med 2007 39 715 721 10.1038/emm.2007.78 18160842

[B41] Wang MJ Lin WW Chen HL Chang YH Ou HC Kuo JS Hong JS Jeng KC Silymarin protects dopaminergic neurons against lipopolysaccharide-induced neurotoxicity by inhibiting microglia activation Eur J Neurosci 2002 16 2103 2112 10.1046/j.1460-9568.2002.02290.x 12473078

[B42] Hou RC Chen HL Tzen JT Jeng KC Effect of sesame antioxidants on LPS-induced NO production by BV2 microglial cells Neuroreport 2003 14 1815 1819 10.1097/00001756-200310060-00011 14534426

[B43] Svensson C Fernaeus SZ Part K Reis K Land T LPS-induced iNOS expression in Bv-2 cells is suppressed by an oxidative mechanism acting on the JNK pathway - a potential role for neuroprotection Brain Res 2010 1322 1 7 20138851 10.1016/j.brainres.2010.01.082

[B44] Kacimi R Giffard RG Yenari MA Endotoxin-activated microglia injure brain derived endothelial cells via NF-kappaB, JAK-STAT and JNK stress kinase pathways J Inflamm 2011 8 7 10.1186/1476-9255-8-7 PMC3061894 21385378

[B45] Wen J Ribeiro R Zhang Y Specific PKC isoforms regulate LPS-stimulated iNOS induction in murine microglial cells J Neuroinflammation 2011 8 38 10.1186/1742-2094-8-38 21510893 PMC3110130

[B46] Maes M Yirmyia R Noraberg J Brene S Hibbeln J Perini G Kubera M Bob P Lerer B Maj M The inflammatory & neurodegenerative (I&ND) hypothesis of depression: leads for future research and new drug developments in depression Metab Brain Dis 2009 24 27 53 10.1007/s11011-008-9118-1 19085093

